# Aurora A Protein Kinase: To the Centrosome and Beyond

**DOI:** 10.3390/biom9010028

**Published:** 2019-01-15

**Authors:** Laura Magnaghi-Jaulin, Grégory Eot-Houllier, Emmanuel Gallaud, Régis Giet

**Affiliations:** University of Rennes, CNRS UMR 6290, IGDR-Institute of Genetics and Development of Rennes, F-35000 Rennes, France; laura.magnaghi@univ-rennes1.fr (L.M.-J.); gregory.eot@univ-rennes1.fr (G.E.-H.); emmanuel.gallaud@univ-rennes1.fr (E.G.)

**Keywords:** Aurora A protein kinase, centrosome, mitotic spindle, polarity, centromere, kinetochore, cohesion, transcription

## Abstract

Accurate chromosome segregation requires the perfect spatiotemporal rearrangement of the cellular cytoskeleton. Isolated more than two decades ago from *Drosophila*, Aurora A is a widespread protein kinase that plays key roles during cell division. Numerous studies have described the localisation of Aurora A at centrosomes, the mitotic spindle, and, more recently, at mitotic centromeres. In this review, we will summarise the cytoskeletal rearrangements regulated by Aurora A during cell division. We will also discuss the recent discoveries showing that Aurora A also controls not only the dynamics of the cortical proteins but also regulates the centromeric proteins, revealing new roles for this kinase during cell division.

## 1. Introduction

Numerous mitotic events are controlled by phosphorylation signalling pathways. In general, extensive phosphorylation events orchestrate the entry into mitosis, whereas waves of dephosphorylation mark the exit [1].

The Aurora kinases are a family of serine/threonine kinases involved in cell-cycle progression, mostly during the G2 and M phases. The founding member of the family, increase in ploidy (IpI1), was isolated three decades ago from budding yeast through a genetic screen to find mutants required for chromosome segregation [2]. In higher eukaryotes, Aurora A was described in flies several years later, after screening a collection of female sterile mutants. Mutations in Aurora A lead to the formation of monopolar spindles in embryos and neural stem cells [3], similar in appearance to the aurora borealis. The name Aurora was then retained as the founding member of the family in higher eukaryotes. Yeast and starfish have one Aurora kinase [2,4,5]. Most animals have at least two and mammals have three Aurora A-like protein kinases, called Aurora A, B and C [6]. All members of this family, at varying degrees of strength, are involved in cancers and have therefore been subjected to the design of anti-cancer molecules [7,8].

Members of the family are evolutionarily conserved and likely share a common ancestor gene, suggested by the presence of a single Aurora gene in starfish [4]. During cell division, Aurora A and B localise to distinct subcellular structures of the mitotic apparatus. Aurora A is located on the duplicated centrosomes and the poles of the mitotic spindle [9,10,11]. Interestingly, it has also been shown to localise to centromeres during mitosis [12], as will be discussed in this review. Aurora B is part of a conserved protein complex (with three other proteins: Inner Centromere Protein (INCENP), Borealin, Survivin) called the chromosomal passenger complex (CPC). The CPC associates with centromeres during mitosis and re-localises to the central spindle and midbody during cytokinesis. Numerous studies and reviews have described a clear role for Aurora B in controlling chromosome condensation, the correction of erroneous chromosome attachment to the mitotic spindle, and cytokinesis [13,14,15]. The function of Aurora C has remained unclear for a long time but recent studies have shown it is essential for the control of meiotic spindle assembly in mammalian oocytes [16,17]. In addition, Aurora C prevents Aurora A from being incorporated into the CPC, a process essential to control meiotic spindle length [18].

**Figure 1:**
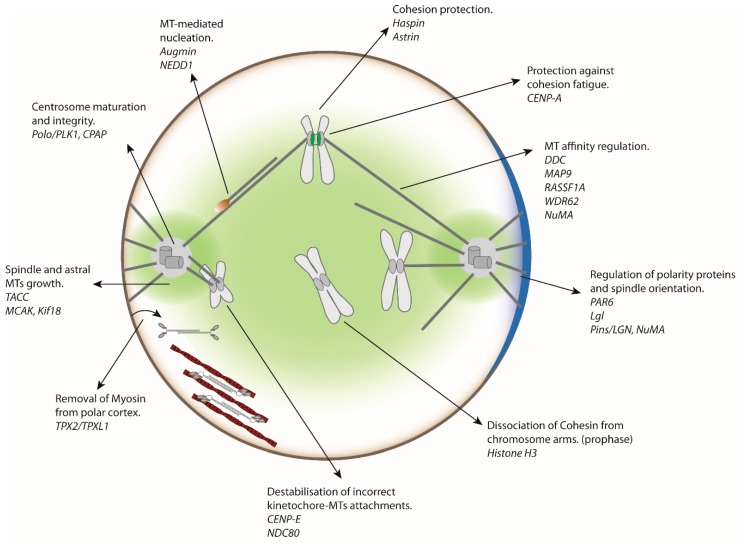
Major substrates of Aurora A protein kinase and their roles during mitosis. The area of influence of Aurora A protein kinase in the cell is indicated in light green. This area includes the cellular cortex, centrosomes and mitotic spindle. Note that a pool of Aurora A is also present at the centromeres. MT: microtubule.

Distinct polar and equatorial mitotic functions have long been assigned to Aurora kinases A and B based on their different cellular localisation [6,19]. However, both enzymes share major structural and sequence similarities. *In cellulo*, their differential targeting to distinct cellular structures is mediated by sequences outside of their catalytic domain, as Aurora kinase domains can be exchanged without affecting their respective localisation or function [20]. In addition, these two kinases have very similar phosphorylation sites in vitro and the use of specific Aurora A and Aurora B inhibitor associated with phospho-proteomic studies has proven that they share identical substrates [21,22]. However, many of these Aurora substrates have not been validated in vivo and it is still not clear if these targets are phosphorylated by Aurora A, Aurora B or both. Moreover, the timing of these phosphorylation events during cell division remains elusive. Finally, their impact on cell division, by mutation analyses has not been systematically investigated. Many reviews have already focused on the regulation of mitosis by Aurora kinase [9,23]. We will, therefore, focus on functional studies of new Aurora A substrates, from entry into mitosis to metaphase/anaphase. In particular, we will highlight Aurora A functions and substrates that participate in the dynamics of cortical proteins, chromosome segregation and centromere-related processes (Figure 1).

## 2. Aurora A Regulates Centrosome Maturation, Centrosome Integrity and Centrosomal Microtubule Polymerisation

Interference with Aurora A by different methods all lead to mitotic spindle assembly defects, ranging from monopolar spindles, with apparently unseparated centrosomes, to bipolar spindles with shorter mitotic spindles. The location of the kinase on the centrosome led to the conclusion that Aurora A regulates centrosome function during mitotic spindle assembly. Indeed, the first studies using monospecific antibodies revealed that a pool of Aurora A protein kinase starts to localise on the duplicated centrosomes, at the end of S phase. In the G2 phase, during the process known as centrosome maturation, the high enrichment of the kinase at the centrosomes coincides with the recruitment of large amounts of pericentriolar material (PCM), an event also known as centrosome maturation [3,11,24,25,26,27,28]. Centrosome maturation is marked by the accumulation of the gamma-tubulin ring complex (γTuRC) on the centrosome, which then promotes extensive microtubule (MT) nucleation [29]. This event is essential, in most cases, to ensure bipolarisation and the correct spatiotemporal assembly of the spindle in mitotic cells. The PCM is composed of many high molecular weight proteins that interact with each other to constitute a larger structure that anchors γTuRC [30,31]. Centrosome maturation studies have been difficult due to the fact that PCM assembly is not linear and some proteins are co-dependent for their recruitment. In addition, PCM assembly and MT nucleation exhibit several differences depending on the model organism and the methods used to investigate their biogenesis. However, studies in several systems, including *Drosophila*, *Xenopus* and human cells, have demonstrated that Aurora A is a key player in the process of centrosome maturation [10,32,33,34,35].

The localisation of Aurora A at centrosomes depends on the presence of a large coil-coiled protein called Spindle-defective protein 2 (Spd-2) (Centrosomal protein 192 (Cep192) in vertebrates), which is recruited to centrioles [32,33]. In *Xenopus*, this large protein displays an Aurora A binding domain and, on the opposite side, a docking site for *Xenopus* Polo like kinase-1 (Plx1). The recruitment of Spd-2 triggers subsequent local enrichment of Aurora A molecules, a process that is sufficient to promote Aurora A dimerization and activation, by trans-auto-phosphorylation of the kinase on a conserved threonine, located in the catalytic domain. Subsequently, Aurora A triggers activation of Plx1 by phosphorylating its activation loop. Plx1 then docks to Spd-2 through Polo boxes on a site previously primed by Cyclin-Dependent Kinase 1 (CDK1)/CyclinB [35]. In humans, the docking of Polo-like kinase 1 (Plk1) to Cep192 is also dependent on the presence of another phosphorylation site (in the absence of Aurora A) and therefore appears to be bi-modal [36]. Strikingly, XlSpd-2 (*Xenopus leavis* Spd-2) does not present binding sites for the direct recruitment of the γTuRC, suggesting the absence of an important factor *in vitro*. Studies in *Drosophila* have provided possible clues about the identity of this missing factor. In this system, DmSpd-2 (*Drosophila melanogaster* Spd-2) is required for the centrosomal accumulation of Centrosomin (Cnn, Cyclin Dependent Kinase 5 Regulatory Subunit-Associated protein 2 (CDK5RAP2) or Centrosomal Protein 215 (Cep215) in mammals), a protein that has an N-terminal binding site for γTuRC [37,38]. Upon entry into mitosis, activated Polo kinase phosphorylates the Cnn/CDK5RAP2 central domain, allowing oligomerisation and its accumulation of the oligomers at the surface of the centrosomal region [39,40]. In addition to recruiting γTuRC, the Cnn/CDK5RAP2/Cep215 N-terminal domain has a binding site for Transforming Acidic Coiled-Coil containing protein (TACC). This recruitment requires direct phosphorylation of TACC phosphorylation by Aurora A [10,41,42]. The main function of TACC, in many model systems, is to bring the MT polymerase Colonic and hepatic Tumour Overexpressed Gene protein (Ch-TOG) into close proximity to the centrosomal region, crucial for its loading onto MTs. Ch-TOG can then stimulate the growth of these newly nucleated astral MTs at the centrosomes [10,42,43].

Therefore, Aurora A contributes through Cnn/CDK5RAP2/Cep215 to the nucleation (via γTuRC) and polymerisation (via TACC/Ch-TOG) of centrosomal MTs.

**Figure 2:**
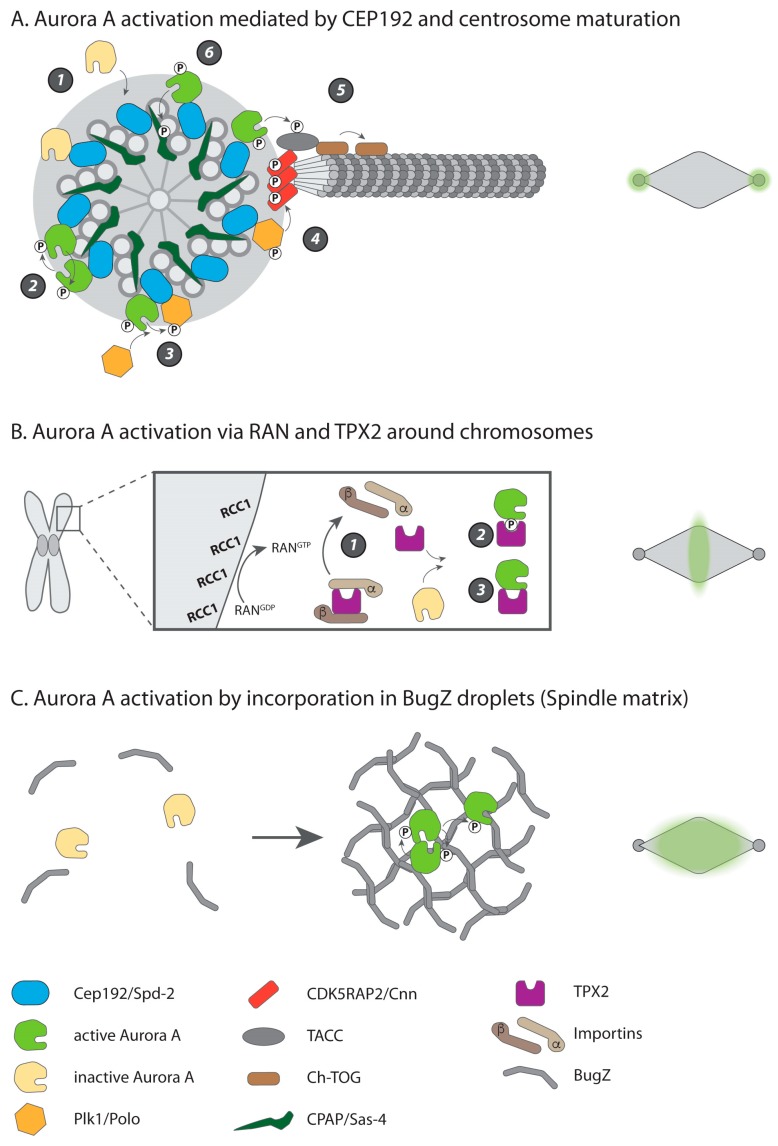
Aurora A activation at the centrosome and the mitotic spindle. (**A**) Model for Aurora A activation and subsequent centrosome maturation based on the literature in different model systems. During mitotic entry, (1) inactive Aurora A is recruited on the centrosome by the Centrosomal Protein 192 (Cep192 or Spd-2 in *Drosophila* and *Caenorhabditis elegans*). (2) This recruitment favours dimerization, trans-autophosphorylation and activation. (3) Polo-like kinase 1 (Plk1) is subsequently phosphorylated by Aurora A and docked on Cep192. (4) Active Plk1 phosphorylates Cyclin-Dependent Kinase 5 Regulatory subunit-Associated Protein 2 (CDK5RAP2) (Cnn in *Drosophila*) that undergoes multimerization. CDK5RAP2 displays a binding site for γ-tubulin ring complex (γTuRC), consequently strong microtubule (MT) nucleation is induced at the centrosome surface. CDK5RAP2 also display a binding site for the Transforming Acidic Coiled-Coil containing Protein 1/Colonic and hepatic Tumour Overexpressed Gene protein (TACC/Ch-TOG) complex. (5) Phosphorylation of TACC by Aurora A triggers recruitment of the TACC/Ch-TOG complex on the centrosome. Ch-TOG stimulates astral MT growth. (6) Aurora A also phosphorylates Centrosomal P4.1 Associated Protein (CPAP), to maintain centrosome architecture. (**B**) Activation of Aurora A around chromosomes by Targeting Protein for Xklp2 (TPX2). (1) The RAs-related Nuclear Guanine Exchange Factor (RAN-GEF) RCC1 is anchored on mitotic chromatin and triggers the formation of a RAN^GTP^ gradient around chromosomes. RAN^GTP^ triggers the release of TPX2 from importins α and β. (2) TPX2 can protect active auto phosphorylated Aurora A from dephosphorylation. (3) TPX2 can bind to and activates Aurora A kinase, without auto phosphorylation. (**C**) Activation of Aurora A by incorporation into droplets. Aurora A can be incorporated into Bub3 interacting and GLEBS motif containing ZNF207 (BugZ) coacervates/droplets (that also contain spindle matrix proteins), to trigger Aurora A dimerization and auto phosphorylation. The right part of the picture shows the location of each Aurora A activated pool (light green).

In addition, Aurora A has an important role in the maintenance of PCM proteins. Indeed, phosphorylation of the Centrosomal P4.1 associated Protein (CPAP) by Aurora A is required to prevent PCM dispersion during M phase [44,45,46]. Overall, Aurora A is a key kinase that orchestrates centrosome assembly and stability, as well as nucleation and polymerisation of centrosomal MTs (Figure 1 and Figure 2A).

## 3. Aurora A Controls Mitotic Spindle Morphogenesis

As soon as the spindle starts to assemble, Aurora A localises to the spindle MTs, in addition to the centrosomes. Strikingly, the spindle-pole-associated Aurora A kinase is activated in a different way from the centrosomal pool. Indeed, the spindle pole localisation and activation of Aurora A is Targeting Protein for Xklp2 (TPX2)-dependent [47,48]. During interphase, TPX2 is retained in the cell nucleus and bound to importins α and β. When the nuclear envelope breaks down (NEBD), the RAs-related Nuclear Guanine Exchange Factor (RAN-GEF) RCC1 (Regulator of Chromosome Condensation 1) bound to mitotic chromatin is activated, leading to the formation of chromatin driven RAN-GTP gradient [49,50]. Such activation triggers the local dissociation of TPX2 from the importins and the local activation of Aurora A. The activation mode of Aurora A by TPX2 has been revealed by several studies. Binding of Aurora A to TPX2 protects the activated kinase from dephosphorylation [51,52]. Interestingly, Aurora A can be activated with the same efficiency, either by autophosphorylation within an Aurora A dimer or after binding to TPX2 [53] (Figure 2B). In human cells, interference with Aurora A-mediated activation by TPX2 produces spindles deprived of spindle-associated Aurora A, whereas the centrosomal pool remains intact [27]. In addition, spindles assembled without centrosomes in *Xenopus* meiotic extracts fail to localise Aurora A and display a low density of MTs, suggesting that Aurora A is involved in spindle MT formation [54].

Interestingly, at least in vitro, the Aurora A dimer is difficult to obtain and displays a high dissociation constant (K_d_) [53]. However, a new exciting mode of activation of Aurora A has been recently described that may help in understanding how this dimer could form at the spindle region: Some proteins, including BugZ (Bub3 interacting and GLEBS motif containing ZNF207), have the property to shift from being soluble into coacervates/droplets and incorporate into the spindle matrix [55,56]. Interestingly Aurora A is able to incorporate at high concentration into these droplets to favour dimerization and auto-activation [57] (Figure 2C).

The spindle pool of Aurora A contributes to spindle MT nucleation by phosphorylating the γTuRC adaptor protein named Neural precursor cell Expressed, Developmentally Down-regulated 1 (NEDD1). NEDD1 contributes to γTuRC targeting to centrosomes and to the mitotic spindle [58,59]. The NEDD1 phosphorylation by Aurora A is required for its recruitment to spindle MTs and efficient MT nucleation. However, this phosphorylation event does not prevent recruitment of NEDD1 to the centrosome [60].

The Augmin octameric complex targets γ-tubulin to pre-existing MTs and is responsible for spindle-dependent MT amplification leading to the formation of branched MTs [61,62,63,64]. Aurora A phosphorylates the Hec1-interacting and centrosome-associated 1 (Hice1) subunit of the Augmin complex. Analysis of phosphomimetic Hice1 mutant showed that Aurora A triggers Hice1 removal from mitotic spindles, leading to a decreased spindle MT density and impaired mitotic progression. In contrast, unphosphorylated Hice1 accumulates prematurely on centrosomal MTs and it was suggested that it may trigger the formation of branched MTs that are not compatible with centrosome separation [65]. Overall, such opposite and not fully understood the regulation of Augmin complex and NEDD1 by Aurora A suggests that tight regulation of MT nucleating activities within spindle sub-compartments are required for mitotic spindle morphogenesis.

At the entry into mitosis, many MT-stabilizing proteins are subjected to phosphorylation events to activate their release from the interphase MT network [66,67,68,69]. Phosphorylation of several Microtubule Associated Proteins (MAP) by Aurora A weakens their binding to MTs in several mitotic spindle compartments. Specifically, an association of the Dynein/Dynactin complex (DDC) with MTs is diminished following phosphorylation at the microtubule binding domain (MBD) of p150*^glued^*, the largest subunit of the Dynactin complex. Consequently, Dynactin and associated Dynein complex strongly accumulate at the spindle poles in Aurora A-deficient cells. In this case, the regulation of MT binding is direct, since the p150*^glued^* MBD, phosphorylated by Aurora A, displays a lower affinity for MTs [70].

Aurora A also regulates MAP9, a protein with MT stabilisation/bundling properties. Phosphorylation of MAP9 by Aurora A prevents proteasome-mediated degradation during cell division, thus contributing to mitotic spindle assembly [71].

Ras association domain-containing protein 1 (RASSF1A) is a tumour suppressor protein that can bind to and stabilise MTs. RASSF1A is phosphorylated by Aurora A on its MBD, resulting in the inhibition of MT binding [72]. Aurora A strongly inhibits the tumour-suppressing properties of RASSF1A, even though the link with MT binding is not clear, because RASSF1A is also an Anaphase-Promoting Complex/Cyclosome (APC/C) inhibitor during early mitosis and therefore controls the degradation of key mitotic targets [72,73,74].

Another known substrate of Aurora A is the MT-binding protein WDR62 (tryptophan (W) aspartic acid (D) Repeat domain 62), of which the gene is involved in microcephaly [75]. Phosphorylation by Aurora A triggers a decrease of WDR62 binding to MTs [76]. Although no functional study has been performed to examine the biological function of phospho-WDR62, a strong genetic interaction between Aurora A and WDR2 has been demonstrated. In addition, the loss of WDR62 causes spindle-assembly defects, spindle assembly checkpoint (SAC)–dependent mitotic delay and aneuploidy, leading ultimately to the loss of neural progenitors [77].

Mitotic Centromere-Associated Kinesin (MCAK) is a protein related to MT-depolymerising-kinesins that localises to centromeres and the spindle poles [78]. This protein is regulated by Aurora B phosphorylation. Aurora B phosphorylation leads to the recruitment of MCAK to mitotic chromatin and the inhibition of its depolymerising activity [79,80,81]. It appears that Aurora B targets MCAK to merotelic kinetochores to resolve incorrect attachments to spindle MTs. Interestingly, in *Xenopus* egg extracts, the spindle pole-associated pool of MCAK is phosphorylated by Aurora A, favouring the displacement of MCAK from the aster centres to the spindle poles and spindle-like structures, probably contributing to the regulation of MT dynamics within spindle sub-compartments [82]. In addition, in human cells, MCAK forms a complex with the depolymerising Kinesin family member 18 (Kif18) at the plus ends of MTs. The MCAK/Kif18 complex is disrupted by Aurora A phosphorylation of MCAK. Inhibition of this complex near centrosomes by Aurora A could contribute to the formation of stable MTs, an event required for correct spindle assembly [83] (Figure 1).

To conclude, many proteins associated with the mitotic spindle are phosphorylated by Aurora A, many of which have not yet been thoroughly characterised. It thus appears complex to integrate the consequences of these different elements of phosphorylation. Antagonistic roles orchestrated by Aurora A are observed on γTuRC recruitment at the mitotic spindle level via concomitant NEDD1 recruitment and Hice1 eviction. It is likely that the role of NEDD1 on the metaphase mitotic spindle is predominant because the mitotic spindles of Aurora A mutants characterised by a lower density of MTs [27,60]. Since aurora A mutants are characterised by a lack of centrosome separation, it is possible that Hice1 eviction on centrosome MTs during the prophase is an essential process to avoid the formation of branched MTs within the two separating asters and thus an ineffective centrosome separation observed in different models [3,26]. The stabilization of MAP9, the recruitment of TACC/Msps (Mini Spindles) and the inactivation of the MCAK/Kif18 complex regulated by Aurora A, are phosphorylation events that contribute to the polymerization of MTs, in accordance with short, less dense astral MTs, observed in Aurora A defective mitotic spindles [10,70,83,84].

## 4. Regulation of Cortical Proteins by Aurora A

Fly neural stem cells (neuroblasts) have proven to be an excellent model to understand the basic principles of cancer stem-cell biology [85,86,87]. Indeed, fly stem cells are polarised and divide asymmetrically to generate a differentiating cell, in which the Notch pathway has to be inhibited, and a self-renewing stem cell, in which the Notch pathway is active. The PAR complex (PAR-6, PAR-3, aPKC, for Partitioning defective 6, Partitioning defective 3, atypical Protein Kinase C, respectively) is a key player in this process. During entry into M phase, the PAR complex accumulates at the apical side and Numb is excluded from the apical cortex. By default, Numb remains at the basal cortex and is inherited by the differentiating cell [86]. The perturbation of PAR accumulation observed in *aurora A* mutants are associated with mis-segregation of Numb, which leads to Notch activation in both daughter cells and tumour formation [88]. Although Aurora A is not detected at the cell cortex, it can promote activation of the PAR complex in fly neuroblasts during entry into mitosis. It does so by phosphorylating the PAR-6 subunit, inducing a conformational change in the complex that triggers a modification of the PAR complex composition and activity. This leads to aPKC-dependent phosphorylation of Numb and its exclusion from the apical cortex [89,90]. Modification of the cortical polarity is also observed in fly epithelia. In this system Lgl (for Lethal giant larvae), a polarity protein required for the maintenance of tissue integrity, is a direct target of Aurora A, allowing correct spindle positioning in the plane of the epithelium [89,90,91,92,93]. *Drosophila* Aurora A also controls spindle orientation in flies by phosphorylating a single amino-acid in the linker domain of Pins (for Partner of Inscuteable, LGN (for leucin glycin asparagin repeats) in mammals). This phosphorylation event triggers the recruitment of the cortical protein Disc large (Dlg), which subsequently binds to Kinesin heavy chain 73 protein located at the plus ends of astral MTs, participating in the correction of spindle orientation defects during telophase [94].

Nuclear Mitotic Apparatus protein 1 (NuMA) is concentrated at spindle poles, where it is required for MT focusing in vertebrates [95,96]. However, a pool of this protein is also present at the cell cortex, where it participates in mitotic spindle orientation by pulling towards astral MTs (for review, reference [97]). The balance between spindle- and cortical-associated NuMA is regulated by Aurora A phosphorylation of its C-terminal domain. Preventing NuMA phosphorylation by Aurora A leads to substantial accumulation of NuMA at the spindle poles and its concomitant loss from the cell cortex leading to mitotic spindle orientation defects [98].

*Drosophila* larval neuroblasts, epithelial cells and cultured human cells are small. The regulation of cortical polarity and orientation machinery at the cortex can be easily explained by the small distance between the centrosome and cell cortex. However, Aurora A also regulates cortical proteins in larger cells. Indeed, Aurora A protein kinase contributes to the cortical clearing of myosin in one-cell *C. elegans* embryos, a process required for the subsequent enrichment of myosin at the equatorial plate of the embryo during cytokinesis. The mechanisms involved in myosin clearing at the spindle poles are still unknown, but require astral MTs, as well as the homologue of the TPX2 protein, TPXL1 (TPX2-like 1) [99].

To conclude, by regulating many cortical and polarity proteins, Aurora A appears to be a key player in regulating cell fate, spindle orientation and the dynamics of myosin during cell division (Figure 1).

## 5. Regulation of Centromeric Proteins by Aurora A

The centromere is a specialized domain of the chromosome required for faithful segregation of sister chromatids during mitosis [100,101,102,103,104,105]. This domain acts as the starting point for the hierarchical building of functional kinetochores. In higher eukaryotes, centromere identity depends on the incorporation of specific nucleosomes containing the Centromere Protein A (CENP-A), a variant of histone H3, exclusively found at centromeres [106,107,108,109,110,111,112]. The centromere propagates epigenetically through cell generations, via the incorporation of CENP-A, which is considered to be an epigenetic mark for centromere inheritance [106,107,108,109,110,111,112]. Centromeres consist of two major distinct structural domains, associated with their specific functions: on one side, the outer-centromere, where the kinetochore is assembled, allows attachment between the kinetochore and MTs of the mitotic spindle; on the other side, the inner-centromere ensures the establishment and protection of cohesion between sister chromatids [113]. Sister chromatid cohesion must persist at the centromeres until the SAC is satisfied and the cell enters anaphase [105,114].

Distinct functions have been assigned to Aurora A and Aurora B because of their different cellular localisation. Indeed, most laboratories describe the contribution of Aurora A to centrosome functions, whereas Aurora B is associated with centromeric activity.

Nonetheless, accumulating evidence over the years suggests that the functions of Aurora A are not confined to centrosomes and that this kinase may play an important role in chromatin-related functions. Indeed, the phosphorylation of histone H3 Threonine 118 (H3T118) along the chromosome arms during prophase is Aurora A-dependent, in agreement with the existence of a soluble and/or a chromatin-associated fraction of Aurora A in the nucleus early in mitosis [115,116]. In addition, the physical presence of an endogenous pool of Aurora A has been detected at centromeres [12], highlighting the existence of an Aurora A-dependent centromeric chromatin pathway.

### 5.1. Role of Aurora A in Kinetochore-Microtubules (KT-MTs) Attachment

The faithful transmission of genetic material during mitosis requires kinetochore attachment to dynamic spindle MTs, followed by chromosome congression towards the equatorial plate. Chromosomes must be correctly bi-oriented in relation to the spindle poles. Back-up mechanisms are involved to avoid erroneous attachment, such as synthelic attachment (both kinetochores are fixed by the same pole) or merotelic attachment (one kinetochore is fixed by MTs linked to both centrosomes) (for a detailed review, [117]). A major role of Aurora B kinase is to correct erroneous attachment between kinetochores and spindle MTs, thus promoting faithful chromosome segregation. However, several studies have shown that defective KT-MTs attachments are corrected not only by a centromere-based mechanism driven by Aurora B, but also a correction mechanism regulated by Aurora A present near the centrosomes [118,119,120]. An increasing number of proteins located near the kinetochore have been shown to be phosphorylated by, or interact with, Aurora A, suggesting a specific centromeric role for this centrosomal kinase. Moreover, recent studies have shown that Aurora A can phosphorylate substrates initially thought to be regulated only by the centromeric kinase, Aurora B, including the Centromere Protein E kinesin (CENP-E). During mitosis, minus-end directed MT transport of mis-attached chromosomes to the centrosomes, is mediated by Dynein allowing Aurora A phosphorylation of the plus end-directed motor protein, CENP-E, near the spindle pole. This phosphorylation results in the dissociation of Protein phosphatase 1 (PP1) from CENP-E and reduces the affinity of CENP-E for MTs [121]. The authors proposed that phosphorylated CENP-E with reduced MT affinity are more prone to bind a stable kinetochore fibre, instead of an isolated dynamic astral MTs. This step could be a prerequisite to bring on CENP-E association to detyrosined spindle MTs [122], and afterwards the progressive sliding of Aurora A-phosphorylated CENP-E along the kinetochore-fibres toward the equator. Away from the Aurora A phosphorylation gradient, CENP-E is then dephosphorylated and re-associates with PP1. The presence of PP1 at the centromere region then counterbalances the destabilization of KT-MTs attachment, induced by the Aurora B phosphorylation gradient at the kinetochores, and contributes to stable attachment of bioriented chromosomes [121].

This model explains well how a polar chromosome can be directed towards the equatorial plate via an already stabilized kinetochore fibre. However, it does not explain why incorrectly attached chromosomes migrate towards the poles to resolve attachment errors. This spatial regulation was considered puzzling, due to a major contribution of the centromeric Aurora B kinase to erroneous KT-MTs attachment [123,124]. The laboratories of Lampson and Maresca solved this discrepancy by demonstrating the involvement of centrosomal Aurora A in the correction of attachment errors in meiosis and mitosis, respectively. Using a system that stabilizes synthelic KT-MTs connections in mitotic *Drosophila* S2 cells, Ye et al. shown that Aurora A overexpression dampens such kinetochore attachment [120]. Then, using various Aurora FRET (Fluorescence Resonance Energy Transfer) sensors, they observed kinetochore phosphorylation induced by an Aurora A kinase gradient emanating from centrosomes when chromosomes were in the vicinity of the spindle poles, whereas Aurora B increased the phosphorylation of unattached kinetochores [120]. Both laboratories established that Aurora A destabilizes KT-MTs attachment near the spindle poles. Moreover, the Mitotic Arrest Deficient 1 protein (Mad1) recruitment near the poles is prevented in the presence of a specific Aurora A inhibitor. The authors also showed that Aurora A is able to phosphorylate serine 55 of the kinetochore protein Nuclear Division Cycle 80 (NDC80), an MT binder regulated by phosphorylation to correct erroneous KT-MTs attachment. Thus, Aurora A gradient contributes to destabilization of mis-attached kinetochores near spindle poles, maybe through phosphorylation of kinetochore substrates, allowing polar ejection forces to reposition the chromosomes at the cell equator and promote opposite kinetochore attachments at the two poles [120].

INCENP is an inner centromeric component of the CPC. Based on the previously described interaction in human cells between Aurora A and INCENP [125], it was shown that overexpression of INCENP is able to induce the targeting of Aurora A to centromeres. This centromere-associated Aurora A pool is involved in chromosome alignment through the phosphorylation of a specific site on NDC80 (serine 69) [126]. Although the mechanistic role is yet to be elucidated, the abolition of such phosphorylation limits oscillations of the bi-oriented chromosome [126]. Thus, distinctly distributed Aurora A proteins promote the differential regulation of NDC80 in KT-MTs connections through a combinatorial phosphorylation mechanism, contributing to chromosomal mobility that ensures their correct alignment on the metaphase plate.

In conclusion, erroneous KT-MTs attachment is corrected by two complementary pathways, depending on chromosome position: One centromeric Aurora B dependent and one centrosomal Aurora A dependent. Overall, the distinct localisation of Aurora A and Aurora B provides fine-tuning of KT-MTs attachment by integrating spatial information of chromosome position. However, recent findings of the presence of an Aurora A pool at centromere opens up new perspectives and interpretations concerning Aurora A dependent regulation of kinetochore and/or centromere substrates.

### 5.2. Aurora A and Chromatin-Associated Transcription Factors during Mitosis

Aurora A kinase activity has recently been implicated in the mitosis-specific inactivation and chromatin dissociation of transcription factors. Mitotic chromatin condensation is not suitable for active transcription, which usually involves the formation of a large multiprotein complex on exposed deoxyribonucleic acid (DNA). Indeed, there is transient and global transcriptional silencing during mitosis. However, many transcription factors remain on the mitotic chromatin and are actively regulated during mitosis. This is true for Yin-Yang 1 (YY1), which specifically interacts with pericentromeric γ-satellite DNA in cycling murine cells. YY1 is a ubiquitously expressed transcription factor which regulates a very large number of target genes involved in cell growth, proliferation, differentiation, metabolism, DNA repair and apoptosis [127,128]. YY1 has been proposed to have a particular potential role in cell division, indeed YY1 regulates transcription at the entry and exit of mitosis, while it dissociates from mitotic chromosomes during mitosis [129]. YY1 is phosphorylated by Aurora A during mitosis, abolishing its DNA binding activity and trans-activating function [129]. Other transcription factors undergo post-translational modifications during mitosis and acquire new mitotic functions. Aurora A phosphorylation of the Runt related transcription factor 3 (RUNX3) promotes its redistribution from chromatin to centrosomes and midbody to regulate chromosome segregation and formation of the mitotic spindle [130].

Another link between Aurora A and transcription to regulate cohesion was highlighted recently. Ataxia Telangiectasia and rad3-Related Protein (ATR), a crucial kinase involved in DNA damage and replication stress responses, localises to centromeres during mitosis. This event is regulated through Aurora A-regulated association with the Centromere Protein F (CENP-F) and transcription products called R loops [131,132]. The authors showed that centromeric ATR co-precipitates with mitotic Aurora A and CENP-F in agreement with the physical presence of Aurora A at the centromeres [12,131]. Activation of ATR by Aurora A locally activates the Checkpoint Kinase 1 (Chk1) and leads to Aurora B activation. ATR and CENP-F are key players in chromosome dynamics, as their inhibition induces whole-chromosome misalignment and premature loss of chromatid cohesion, respectively [131,133]. Because the interaction between CENP-F and ATR is under the control of Aurora A, the kinase may also prevent dissociation of cohesion through ATR and CENP-F.

### 5.3. Role of Aurora A in the Maintenance of Sister Chromatid Cohesion

Two steps are necessary to achieve chromatid cohesion in vertebrates during mitosis: (i) during prophase, Cohesins dissociate from the chromosome arms, under the control of the Plk1 and Aurora B kinases (prophase pathway), whereas cohesion is maintained at the centromere; (ii) during the metaphase/anaphase transition, activation of the APC/C complex induces the degradation of Securin and Cyclin B, resulting in the activation of Separase, which in turn can cleave residual Cohesin at the centromere and trigger separation of the sister chromatids. Decoupling of the two steps is ensured at the centromeres by the Cohesin protector Shugoshin 1 (Sgo1), which recruits the Protein Phosphatase 2A (PP2A) to counterbalance kinase-induced-dissociation during the prophase pathway [134]. Finally, the second step must be triggered simultaneously for all chromosomes to achieve correct chromosome segregation and is under the control of the SAC. In addition, cells must also prevent cohesion fatigue, a phenomenon that leads to uncoordinated sister chromatid disjunction under sustained spindle tension [135,136].

**Figure 3:**
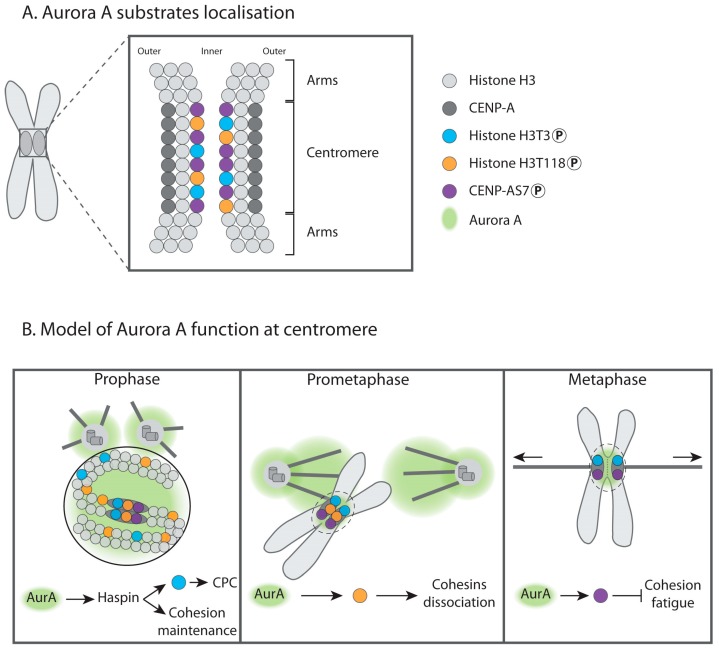
Histone phosphorylation events are controlled by Aurora A during mitosis. (**A**) Representation of an enlarged centromere showing nucleosomes (coloured circles). A nucleosome containing phosphorylated Threonine 3 of histone H3 (H3T3) is shown in blue, phosphorylated Threonine 118 of histone H3 (H3T118) is shown in orange. A nucleosome containing the centromere-specific phosphorylated Serine 7 of CENP-A (CENP-AS7) is displayed in purple. The light and dark grey circles represent histone H3 and CENP-A containing nucleosomes respectively. (**B**) Centromeric histone phosphorylations by Aurora A during mitosis and possible functions. During prophase and prometaphase, Aurora A (AurA, green) phosphorylates Haspin, a kinase required for cohesion maintenance and H3T3 phosphorylation at centromeres, necessary for CPC assembly. Aurora A also phosphorylates H3T118 on chromosome arms but mostly at centromeres. This phosphorylation may create a chromatin environment that promotes Cohesin dissociation. CENP-AS7 is phosphorylated at centromeres only. We speculate that the centrosomal pool of Aurora A is responsible for H3T118 phosphorylation because unaligned chromosomes in the vicinity of the centrosomes display more intense H3T118 signal (Middle panel). In metaphase, when chromosomes are aligned at the equator plate, phosphorylated H3T3 and CENP-AS7 are still present at the centromere, while phosphorylation of H3-T118 is lost. When sister chromatids are under tension, phosphorylation of CENPA-S7A by a centromeric pool of Aurora A (surrounded by the dashed line) prevents cohesion fatigue (Right panel).

In the last decade, accumulating evidence has suggested that Aurora A plays an unexpected role during cell division including the regulation of mitosis duration, by promoting the degradation of Cyclin B [137]. Recently, Yu F et al., (2017) showed that Aurora A phosphorylates mitotic Haspin kinase at multiple sites during the late G2 phase and modulates its activity [138]. Haspin is necessary for assembly of the CPC at the inner centromere, through histone H3 Threonine 3 (H3T3) phosphorylation; moreover, Haspin binds to the Cohesin regulatory subunit named Precocious dissociation of sisters 5B (Pds5B). This interaction is required to retain Cohesins at the centromeres to protect sister-chromatid cohesion until the onset of anaphase. Disruption of the Haspin/Pds5B interaction causes premature sister chromatid separation (PSCS) [139]. Further studies will be necessary to characterise the precise functions of Aurora A-phosphorylated residues of the Haspin kinase (Figure 1 and Figure 3).

Another kinetochore protein regulated by Aurora A kinase activity and involved in the protection of chromatid cohesion is Astrin [140]. Phosphorylation of Astrin by Aurora A enables its interaction with many of its mitotic partners, such as Securin. Astrin localises to bi-oriented sister kinetochores prior to the onset of anaphase. Moreover, Astrin phosphorylation by Aurora A promotes its interaction with Securin, leading to its ubiquitination, degradation, and subsequently inactivation of Separase, maintaining the cohesion of sister chromatids. Preventing Astrin phosphorylation hampers the inactivation of Separase, leading to PSCS and delaying mitosis following activation of the SAC [141].

Strikingly, Aurora A can phosphorylate histone H3 at position T118. *In vitro*, such phosphorylation weakens histone DNA-contacts, disrupting the nucleosome structure [142]. This phosphorylation event occurs at centromeres and chromosome arms during prophase and is lost upon chromosome alignment during metaphase but remains at the centromeres of misaligned chromosomes [116]. Phosphorylation of H3 at T118 takes place at the inner part of the centromere. An increase in Aurora A activity leads to increased H3T118 phosphorylation, causing the loss of cohesion and reduced levels of Cohesin and Condensin I on the chromatin. The authors suggested that the status of Aurora A-dependent H3T118 phosphorylation could play an important role in organizing chromatin structure near centromeres to acquire suitable levels of Cohesin and Condensin I to allow conformational flexibility of misaligned chromosomes for MT attachment. Once sister kinetochores become bioriented and tension is sensed, phosphorylated H3T118 is removed to allow a higher degree of centromere rigidity [116] (Figure 3).

CENP-A is a histone H3 variant found in the centromere region. A recent study demonstrated the role of Aurora A in the phosphorylation of the Serine 7 of CENP-A (CENP-AS7) at the inner centromeres of sister chromatids [12]. Interference with Aurora A or expression of a non-phosphorylatable version of CENP-AS7 induces premature loss of sister-chromatid cohesion and leads to the partial removal of Sgo1 from the centromeres of separated sister chromatids [12]. However, Sgo1 loss from centromeres is unlikely to be the sole cause of cohesion defects, as Cohesin depletion does not lead to Sgo1 removal from separated chromatids. Moreover, non-phosphorylable CENP-AS7A mutant cells still recruit Sgo1 to centromeres, excluding then a direct interaction between phosphorylated CENP-A nucleosomes and Sgo1. The inter-kinetochore distance is under the control of the phosphorylation status of CENP-AS7 and mitotic chromatin fibres containing phosphorylated CENP-AS7 stretch more easily than those containing non-phosphorylated CENP-A. Thus, the authors proposed that Aurora A-dependent phosphorylation of CENP-AS7 may modify inner centromere chromatin plasticity, inducing the consolidation of Sgo1 at centromeres during the short time window preceding entry into anaphase, providing the first regulated protection mechanism against fatigue until the last chromosome is bioriented [12]. This mechanism against fatigue does not appear to exist in lower eukaryotes, such as yeast, which harbours only one Aurora kinase, since tension leads to Sgo1 homologue eviction in *Saccharomyces cerevisiae* [143]. It is therefore conceivable that during the course of evolution, higher eukaryotes developed additional mechanisms that increase the accuracy of chromosome segregation by actively protecting sister chromatid cohesion against the fatigue induced by spindle forces. Aurora A recruitment to centromeres allows CENP-AS7 phosphorylation, thus acting as a final “safety switch” to keep the sister chromatids attached until all chromosomes are ready to initiate anaphase (Figure 1 and Figure 3).

An attractive hypothesis is that the Aurora A-dependent control of sister chromatid cohesion is a two-step process regulated in space and time by different histone modifications. First, phosphorylation of H3T118 appears to be essential for the organization of chromatin structure to achieve a favourable chromatin environment for the dissociation of Cohesins from the chromosomes [116]. Once biorientation takes place and tension is sensed [143,144,145], phosphorylated H3T118 is removed to allow a higher degree of centromere rigidity to counterbalance opposite spindle forces [116].

However, the amphitelic attachment of MTs to kinetochores, which induces chromosome biorientation, does not simultaneously occur for all chromosomes and cohesion must be protected while free kinetochores are being attached. Thus, to counterbalance the loss of phosphorylated-H3T118-dependent chromatin rigidity, the centromeric pool of Aurora A, responsible for CENP-AS7 nucleosomes, may promote chromatin flexibility to prevent premature abolition of centromeric cohesion by promoting the consolidation of Sgo1 until the last chromosome is bioriented and the criteria of the SAC are satisfied [12]. As this function is required when the chromosomes are far from the spindle poles, it is possible that the pool of Aurora A at the centromeres, amassed via its interaction with INCENP in a Bub1-dependent manner, is responsible for such phosphorylation [12,126]. In conclusion, based on our current state of knowledge, Aurora A: (i) Activates Haspin kinase, which prevents the premature dissociation of Cohesin and phosphorylates H3T3 to recruit the CPC; (ii) controls Cohesin occupancy by the phosphorylation of H3T118; (iii) and phosphorylates CENP-AS7 to stabilizes Sgo1 after chromosome biorientation to prevent cohesion fatigue. Aurora A is thus a linchpin of an elaborate centromeric histone modification landscape that drives chromosome cohesion regulation circuits during mitosis (Figure 3).

## 6. Conclusions

Aurora A has been studied by scientists for many years because of its close link to cancer. Its highly visible location in centrosomes, organising elements of the mitotic spindle, led to the initial conclusion that its role is limited to organisation of the mitotic spindle. However, it appears that activation of this protein is actually much more complex than was first imagined. Indeed, various activators coexist in the cell, probably resulting in the specificity of the phosphorylation of substrates in time and space. Indeed, it is clear that a gradient of this protein kinase is able to modify the properties of the cellular cortex. Surprisingly, chromatin and the chromosomes themselves, considered to be the private garden of Aurora B and CPC, are accessible to the Aurora A kinase, either directly through an active pool of this kinase present in the centromeres or when the chromatin is close to the centrosomes. Further studies will be needed to better understand how Aurora A is regulated. It is likely that surprising targets and unexpected functions are yet to be identified.

## References

[1] Nigg E.A. (2001). Mitotic kinases as regulators of cell division and its checkpoints. Nat. Rev. Mol. Cell Biol..

[2] Francisco L., Chan C.S. (1994). Regulation of yeast chromosome segregation by Ipl1 protein kinase and type 1 protein phosphatase. Cell. Mol. Biol. Res..

[3] Glover D.M., Leibowitz M.H., McLean D.A., Parry H. (1995). Mutations in *aurora* prevent centrosome separation leading to the formation of monopolar spindles. Cell.

[4] Abe Y., Okumura E., Hosoya T., Hirota T., Kishimoto T. (2010). A single starfish Aurora kinase performs the combined functions of Aurora-A and Aurora-B in human cells. J. Cell Sci..

[5] Petersen J., Hagan I.M. (2003). *S. Pombe* aurora kinase/survivin is required for chromosome condensation and the spindle checkpoint attachment response. Curr. Biol..

[6] Carmena M., Earnshaw W.C. (2003). The cellular geography of aurora kinases. Nat. Rev. Mol. Cell Biol..

[7] Carvajal R.D., Tse A., Schwartz G.K. (2006). Aurora kinases: New targets for cancer therapy. Clin. Cancer Res..

[8] Tang A., Gao K., Chu L., Zhang R., Yang J., Zheng J. (2017). Aurora kinases: Novel therapy targets in cancers. Oncotarget.

[9] Barr A.R., Gergely F. (2007). Aurora-A: The maker and breaker of spindle poles. J. Cell Sci..

[10] Giet R., McLean D., Descamps S., Lee M.J., Raff J.W., Prigent C., Glover D.M. (2002). Drosophila aurora A kinase is required to localize D-TACC to centrosomes and to regulate astral microtubules. J. Cell Biol..

[11] Roghi C., Giet R., Uzbekov R., Morin N., Chartrain I., Le Guellec R., Couturier A., Doree M., Philippe M., Prigent C. (1998). The Xenopus protein kinase pEg2 associates with the centrosome in a cell cycle-dependent manner, binds to the spindle microtubules and is involved in bipolar mitotic spindle assembly. J. Cell Sci..

[12] Eot-Houllier G., Magnaghi-Jaulin L., Fulcrand G., Moyroud F.X., Monier S., Jaulin C. (2018). Aurora A-dependent CENP-A phosphorylation at inner centromeres protects bioriented chromosomes against cohesion fatigue. Nat. Commun..

[13] Afonso O., Figueiredo A.C., Maiato H. (2017). Late mitotic functions of aurora kinases. Chromosoma.

[14] Carmena M., Wheelock M., Funabiki H., Earnshaw W.C. (2012). The chromosomal passenger complex (CPC): From easy rider to the godfather of mitosis. Nat. Rev. Mol. Cell Biol..

[15] Trivedi P., Stukenberg P.T. (2015). A centromere-signaling network underlies the coordination among mitotic events. Trends Biochem. Sci..

[16] Yang K.T., Tang C.J., Tang T.K. (2015). Possible role of aurora-C in meiosis. Front. Oncol..

[17] Balboula A.Z., Nguyen A.L., Gentilello A.S., Quartuccio S.M., Drutovic D., Solc P., Schindler K. (2016). Haspin kinase regulates microtubule-organizing center clustering and stability through aurora kinase C in mouse oocytes. J. Cell Sci..

[18] Nguyen A.L., Drutovic D., Vazquez B.N., El Yakoubi W., Gentilello A.S., Malumbres M., Solc P., Schindler K. (2018). Genetic interactions between the aurora kinases reveal new requirements for AURKB and AURKC during oocyte meiosis. Curr. Biol..

[19] Carmena M., Ruchaud S., Earnshaw W.C. (2009). Making the auroras glow: Regulation of aurora A and B kinase function by interacting proteins. Curr. Opin. Cell Biol..

[20] Li S., Deng Z., Fu J., Xu C., Xin G., Wu Z., Luo J., Wang G., Zhang S., Zhang B. (2015). Spatial compartmentalization specializes the function of aurora A and aurora B. J. Biol. Chem..

[21] Ferrari S., Marin O., Pagano M.A., Meggio F., Hess D., El-Shemerly M., Krystyniak A., Pinna L.A. (2005). Aurora-A site specificity: A study with synthetic peptide substrates. Biochem. J..

[22] Kettenbach A.N., Schweppe D.K., Faherty B.K., Pechenick D., Pletnev A.A., Gerber S.A. (2011). Quantitative phosphoproteomics identifies substrates and functional modules of aurora and Polo-like kinase activities in mitotic cells. Sci. Signal.

[23] Giet R., Petretti C., Prigent C. (2005). Aurora kinases, aneuploidy and cancer, a coincidence or a real link?. Trends Cell Biol..

[24] Berdnik D., Knoblich J.A. (2002). Drosophila aurora-a is required for centrosome maturation and actin-dependent asymmetric protein localization during mitosis. Curr. Biol..

[25] Giet R., Prigent C. (2001). The non-catalytic domain of the *Xenopus laevis* auroraA kinase localises the protein to the centrosome. J. Cell Sci..

[26] Hannak E., Kirkham M., Hyman A.A., Oegema K. (2001). Aurora-A kinase is required for centrosome maturation in *Caenorhabditis elegans*. J. Cell Biol..

[27] Kufer T.A., Sillje H.H., Korner R., Gruss O.J., Meraldi P., Nigg E.A. (2002). Human TPX2 is required for targeting aurora-A kinase to the spindle. J. Cell Biol..

[28] Schumacher J.M., Ashcroft N., Donovan P.J., Golden A. (1998). A highly conserved centrosomal kinase, AIR-1, is required for accurate cell cycle progression and segregation of developmental factors in Caenorhabditis elegans embryos. Development.

[29] Tillery M.M.L., Blake-Hedges C., Zheng Y., Buchwalter R.A., Megraw T.L. (2018). Centrosomal and non-centrosomal microtubule-organizing centers (MTOCs) in *Drosophila melanogaster*. Cells.

[30] Muller H., Schmidt D., Steinbrink S., Mirgorodskaya E., Lehmann V., Habermann K., Dreher F., Gustavsson N., Kessler T., Lehrach H. (2010). Proteomic and functional analysis of the mitotic *Drosophila* centrosome. EMBO J..

[31] Reboutier D., Troadec M.B., Cremet J.Y., Fukasawa K., Prigent C. (2012). Nucleophosmin/B23 activates aurora A at the centrosome through phosphorylation of serine 89. J. Cell Biol..

[32] Joukov V., De Nicolo A., Rodriguez A., Walter J.C., Livingston D.M. (2010). Centrosomal protein of 192 kDa (Cep192) promotes centrosome-driven spindle assembly by engaging in organelle-specific aurora A activation. Proc. Natl. Acad. Sci. USA.

[33] Pelletier L., Ozlu N., Hannak E., Cowan C., Habermann B., Ruer M., Muller-Reichert T., Hyman A.A. (2004). The *Caenorhabditis elegans* centrosomal protein SPD-2 is required for both pericentriolar material recruitment and centriole duplication. Curr. Biol..

[34] Zhu F., Lawo S., Bird A., Pinchev D., Ralph A., Richter C., Muller-Reichert T., Kittler R., Hyman A.A., Pelletier L. (2008). The mammalian SPD-2 ortholog CEP192 regulates centrosome biogenesis. Curr. Biol..

[35] Joukov V., Walter J.C., De Nicolo A. (2014). The CEP192-organized aurora A-Plk1 cascade is essential for centrosome cycle and bipolar spindle assembly. Mol. Cell.

[36] Meng L., Park J.E., Kim T.S., Lee E.H., Park S.Y., Zhou M., Bang J.K., Lee K.S. (2015). Bimodal interaction of mammalian Polo-like kinase 1 and a centrosomal scaffold, CEP192, in the regulation of bipolar spindle formation. Mol. Cell Biol..

[37] Fong K.W., Choi Y.K., Rattner J.B., Qi R.Z. (2008). CDK5RAP2 is a pericentriolar protein that functions in centrosomal attachment of the γ-tubulin ring complex. Mol. Biol. Cell.

[38] Zhang J., Megraw T.L. (2007). Proper recruitment of γ-tubulin and D-TACC/Msps to embryonic *Drosophila* centrosomes requires centrosomin Motif 1. Mol. Biol. Cell.

[39] Conduit P.T., Feng Z., Richens J.H., Baumbach J., Wainman A., Bakshi S.D., Dobbelaere J., Johnson S., Lea S.M., Raff J.W. (2014). The centrosome-specific phosphorylation of Cnn by Polo/Plk1 drives Cnn scaffold assembly and centrosome maturation. Dev. Cell.

[40] Conduit P.T., Raff J.W. (2015). Different *Drosophila* cell types exhibit differences in mitotic centrosome assembly dynamics. Curr. Biol..

[41] Barros T.P., Kinoshita K., Hyman A.A., Raff J.W. (2005). Aurora A activates D-TACC-Msps complexes exclusively at centrosomes to stabilize centrosomal microtubules. J. Cell Biol..

[42] Kinoshita K., Noetzel T.L., Pelletier L., Mechtler K., Drechsel D.N., Schwager A., Lee M., Raff J.W., Hyman A.A. (2005). Aurora A phosphorylation of TACC3/Maskin is required for centrosome-dependent microtubule assembly in mitosis. J. Cell Biol..

[43] Lee M.J., Gergely F., Jeffers K., Peak-Chew S.Y., Raff J.W. (2001). Msps/Xmap215 interacts with the centrosomal protein D-TACC to regulate microtubule behaviour. Nat. Cell Biol..

[44] Chou E.J., Hung L.Y., Tang C.J., Hsu W.B., Wu H.Y., Liao P.C., Tang T.K. (2016). Phosphorylation of CPAP by aurora-A maintains spindle pole integrity during mitosis. Cell Rep..

[45] De Luca M., Brunetto L., Asteriti I.A., Giubettini M., Lavia P., Guarguaglini G. (2008). Aurora-A and ch-TOG act in a common pathway in control of spindle pole integrity. Oncogene.

[46] Marumoto T., Honda S., Hara T., Nitta M., Hirota T., Kohmura E., Saya H. (2003). Aurora-A kinase maintains the fidelity of early and late mitotic events in Hela cells. J. Biol. Chem..

[47] Wittmann T., Boleti H., Antony C., Karsenti E., Vernos I. (1998). Localization of the kinesin-like protein Xklp2 to spindle poles requires a leucine zipper, a microtubule-associated protein, and dynein. J. Cell Biol..

[48] Wittmann T., Wilm M., Karsenti E., Vernos I. (2000). Tpx2, a novel Xenopus map involved in spindle pole organization. J. Cell Biol..

[49] Carazo-Salas R.E., Gruss O.J., Mattaj I.W., Karsenti E. (2001). Ran-GTP coordinates regulation of microtubule nucleation and dynamics during mitotic-spindle assembly. Nat. Cell Biol..

[50] Carazo-Salas R.E., Guarguaglini G., Gruss O.J., Segref A., Karsenti E., Mattaj I.W. (1999). Generation of GTP-bound ran by RCC1 is required for chromatin-induced mitotic spindle formation. Nature.

[51] Bayliss R., Sardon T., Vernos I., Conti E. (2003). Structural basis of aurora-A activation by TPX2 at the mitotic spindle. Mol. Cell.

[52] Eyers P.A., Erikson E., Chen L.G., Maller J.L. (2003). A novel mechanism for activation of the protein kinase aurora A. Curr. Biol..

[53] Zorba A., Buosi V., Kutter S., Kern N., Pontiggia F., Cho Y.J., Kern D. (2014). Molecular mechanism of aurora A kinase autophosphorylation and its allosteric activation by TPX2. Elife.

[54] Tsai M.Y., Wiese C., Cao K., Martin O., Donovan P., Ruderman J., Prigent C., Zheng Y. (2003). A Ran signalling pathway mediated by the mitotic kinase aurora A in spindle assembly. Nat. Cell Biol..

[55] Jiang H., He X., Wang S., Jia J., Wan Y., Wang Y., Zeng R., Yates J., Zhu X., Zheng Y. (2014). A microtubule-associated zinc finger protein, BuGZ, regulates mitotic chromosome alignment by ensuring Bub3 stability and kinetochore targeting. Dev. Cell.

[56] Jiang H., Wang S., Huang Y., He X., Cui H., Zhu X., Zheng Y. (2015). Phase transition of spindle-associated protein regulate spindle apparatus assembly. Cell.

[57] Huang Y., Li T., Ems-McClung S.C., Walczak C.E., Prigent C., Zhu X., Zhang X., Zheng Y. (2018). Aurora A activation in mitosis promoted by BuGZ. J. Cell Biol..

[58] Haren L., Remy M.H., Bazin I., Callebaut I., Wright M., Merdes A. (2006). Nedd1-dependent recruitment of the γ-tubulin ring complex to the centrosome is necessary for centriole duplication and spindle assembly. J. Cell Biol..

[59] Luders J., Patel U.K., Stearns T. (2006). GCP-WD is a γ-tubulin targeting factor required for centrosomal and chromatin-mediated microtubule nucleation. Nat. Cell Biol..

[60] Pinyol R., Scrofani J., Vernos I. (2013). The role of NEDD1 phosphorylation by aurora A in chromosomal microtubule nucleation and spindle function. Curr. Biol..

[61] Goshima G., Mayer M., Zhang N., Stuurman N., Vale R.D. (2008). Augmin: A protein complex required for centrosome-independent microtubule generation within the spindle. J. Cell Biol..

[62] Petry S., Groen A.C., Ishihara K., Mitchison T.J., Vale R.D. (2013). Branching microtubule nucleation in Xenopus egg extracts mediated by augmin and TPX2. Cell.

[63] Uehara R., Nozawa R.S., Tomioka A., Petry S., Vale R.D., Obuse C., Goshima G. (2009). The augmin complex plays a critical role in spindle microtubule generation for mitotic progression and cytokinesis in human cells. Proc. Natl. Acad. Sci. USA.

[64] Wu G., Lin Y.T., Wei R., Chen Y., Shan Z., Lee W.H. (2008). Hice1, a novel microtubule-associated protein required for the maintenance of spindle integrity and chromosomal stability in human cells. Mol. Cell Biol..

[65] Tsai C.Y., Ngo B., Tapadia A., Hsu P.H., Wu G., Lee W.H. (2011). Aurora-A phosphorylates augmin complex component Hice1 protein at an N-terminal serine/threonine cluster to modulate its microtubule binding activity during spindle assembly. J. Biol. Chem..

[66] McHedlishvili N., Matthews H.K., Corrigan A., Baum B. (2018). Two-step interphase microtubule disassembly aids spindle morphogenesis. BMC Biol..

[67] Petrone A., Adamo M.E., Cheng C., Kettenbach A.N. (2016). Identification of candidate cyclin-dependent kinase 1 (Cdk1) substrates in mitosis by quantitative phosphoproteomics. Mol. Cell Proteom..

[68] Verde F., Dogterom M., Stelzer E., Karsenti E., Leibler S. (1992). Control of microtubule dynamics and length by cyclin A- and cyclin B-dependent kinases in xenopus egg extracts. J. Cell Biol..

[69] Verde F., Labbe J.C., Doree M., Karsenti E. (1990). Regulation of microtubule dynamics by CDC2 protein kinase in cell-free extracts of Xenopus eggs. Nature.

[70] Rome P., Montembault E., Franck N., Pascal A., Glover D.M., Giet R. (2010). Aurora A contributes to p150(glued) phosphorylation and function during mitosis. J. Cell Biol..

[71] Venoux M., Basbous J., Berthenet C., Prigent C., Fernandez A., Lamb N.J., Rouquier S. (2008). ASAP is a novel substrate of the oncogenic mitotic kinase aurora-A: Phosphorylation on Ser625 is essential to spindle formation and mitosis. Hum. Mol. Genet..

[72] Rong R., Jiang L.Y., Sheikh M.S., Huang Y. (2007). Mitotic kinase aurora-A phosphorylates RASSF1A and modulates RASSF1A-mediated microtubule interaction and M-phase cell cycle regulation. Oncogene.

[73] Chow C., Wong N., Pagano M., Lun S.W., Nakayama K.I., Nakayama K., Lo K.W. (2012). Regulation of APC/CCdc20 activity by RASSF1A-APC/CCDC20 circuitry. Oncogene.

[74] Song S.J., Kim S.J., Song M.S., Lim D.S. (2009). Aurora B-mediated phosphorylation of RASSF1A maintains proper cytokinesis by recruiting syntaxin16 to the midzone and midbody. Cancer Res..

[75] Yu T.W., Mochida G.H., Tischfield D.J., Sgaier S.K., Flores-Sarnat L., Sergi C.M., Topcu M., McDonald M.T., Barry B.J., Felie J.M. (2010). Mutations in *WDR62*, encoding a centrosome-associated protein, cause microcephaly with simplified gyri and abnormal cortical architecture. Nat. Genet..

[76] Lim N.R., Yeap Y.Y., Zhao T.T., Yip Y.Y., Wong S.C., Xu D., Ang C.S., Williamson N.A., Xu Z., Bogoyevitch M.A. (2015). Opposing roles for JNK and aurora A in regulating the association of wdr62 with spindle microtubules. J. Cell Sci..

[77] Chen J.F., Zhang Y., Wilde J., Hansen K.C., Lai F., Niswander L. (2014). Microcephaly disease gene WDR62 regulates mitotic progression of embryonic neural stem cells and brain size. Nat. Commun..

[78] Wordeman L., Mitchison T.J. (1995). Identification and partial characterization of mitotic centromere-associated kinesin, a kinesin-related protein that associates with centromeres during mitosis. J. Cell Biol..

[79] Andrews P.D., Ovechkina Y., Morrice N., Wagenbach M., Duncan K., Wordeman L., Swedlow J.R. (2004). Aurora B regulates MCAK at the mitotic centromere. Dev. Cell.

[80] Knowlton A.L., Lan W., Stukenberg P.T. (2006). Aurora B is enriched at merotelic attachment sites, where it regulates MCAK. Curr. Biol..

[81] Lan W., Zhang X., Kline-Smith S.L., Rosasco S.E., Barrett-Wilt G.A., Shabanowitz J., Hunt D.F., Walczak C.E., Stukenberg P.T. (2004). Aurora B phosphorylates centromeric MCAK and regulates its localization and microtubule depolymerization activity. Curr. Biol..

[82] Zhang X., Ems-McClung S.C., Walczak C.E. (2008). Aurora A phosphorylates MCAK to control ran-dependent spindle bipolarity. Mol. Biol. Cell.

[83] Tanenbaum M.E., Macurek L., van der Vaart B., Galli M., Akhmanova A., Medema R.H. (2011). A complex of Kif18b and MCAK promotes microtubule depolymerization and is negatively regulated by aurora kinases. Curr. Biol..

[84] Reboutier D., Troadec M.B., Cremet J.Y., Chauvin L., Guen V., Salaun P., Prigent C. (2013). Aurora A is involved in central spindle assembly through phosphorylation of Ser19 in p150glued. J. Cell Biol..

[85] Gonzalez C. (2007). Spindle orientation, asymmetric division and tumour suppression in *Drosophila* stem cells. Nat. Rev. Genet..

[86] Knoblich J.A. (2010). Asymmetric cell division: Recent developments and their implications for tumour biology. Nat. Rev. Mol. Cell Biol..

[87] Siller K.H., Doe C.Q. (2009). Spindle orientation during asymmetric cell division. Nat. Cell Biol..

[88] Wang H., Somers G.W., Bashirullah A., Heberlein U., Yu F., Chia W. (2006). Aurora-A acts as a tumor suppressor and regulates self-renewal of *Drosophila* neuroblasts. Genes Dev..

[89] Lee C.Y., Andersen R.O., Cabernard C., Manning L., Tran K.D., Lanskey M.J., Bashirullah A., Doe C.Q. (2006). *Drosophila* aurora-A kinase inhibits neuroblast self-renewal by regulating aPKC/Numb cortical polarity and spindle orientation. Genes Dev..

[90] Wirtz-Peitz F., Nishimura T., Knoblich J.A. (2008). Linking cell cycle to asymmetric division: Aurora-A phosphorylates the par complex to regulate numb localization. Cell.

[91] Bell G.P., Fletcher G.C., Brain R., Thompson B.J. (2015). Aurora kinases phosphorylate Lgl to induce mitotic spindle orientation in *Drosophila* epithelia. Curr. Biol..

[92] Carvalho C.A., Moreira S., Ventura G., Sunkel C.E., Morais-de-Sa E. (2015). Aurora a triggers Lgl cortical release during symmetric division to control planar spindle orientation. Curr. Biol..

[93] Nakajima Y., Gibson M.C. (2015). Epithelial cell division: Aurora kicks Lgl to the cytoplasmic curb. Curr. Biol..

[94] Johnston C.A., Hirono K., Prehoda K.E., Doe C.Q. (2009). Identification of an aurora-A/Pins^LINKER^/Dlg spindle orientation pathway using induced cell polarity in S2 cells. Cell.

[95] Merdes A., Heald R., Samejima K., Earnshaw W.C., Cleveland D.W. (2000). Formation of spindle poles by dynein/dynactin-dependent transport of NuMA. J. Cell Biol..

[96] Merdes A., Ramyar K., Vechio J.D., Cleveland D.W. (1996). A complex of NuMA and cytoplasmic dynein is essential for mitotic spindle assembly. Cell.

[97] di Pietro F., Echard A., Morin X. (2016). Regulation of mitotic spindle orientation: An integrated view. EMBO Rep..

[98] Gallini S., Carminati M., De Mattia F., Pirovano L., Martini E., Oldani A., Asteriti I.A., Guarguaglini G., Mapelli M. (2016). NuMa phosphorylation by aurora-a orchestrates spindle orientation. Curr. Biol..

[99] Mangal S., Sacher J., Kim T., Osorio D.S., Motegi F., Carvalho A.X., Oegema K., Zanin E. (2018). TPXL-1 activates aurora A to clear contractile ring components from the polar cortex during cytokinesis. J. Cell Biol..

[100] Bloom K., Joglekar A. (2010). Towards building a chromosome segregation machine. Nature.

[101] Fitzgerald-Hayes M. (1987). Yeast centromeres. Yeast.

[102] Verdaasdonk J.S., Bloom K. (2011). Centromeres: Unique chromatin structures that drive chromosome segregation. Nat. Rev. Mol. Cell Biol..

[103] Westhorpe F.G., Straight A.F. (2014). The centromere: Epigenetic control of chromosome segregation during mitosis. Cold Spring Harb. Perspect. Biol..

[104] Willard H.F. (1990). Centromeres of mammalian chromosomes. Trends Genet..

[105] Cleveland D.W., Mao Y., Sullivan K.F. (2003). Centromeres and kinetochores: From epigenetics to mitotic checkpoint signaling. Cell.

[106] Allshire R.C., Karpen G.H. (2008). Epigenetic regulation of centromeric chromatin: Old dogs, new tricks?. Nat. Rev. Genet..

[107] Bernad R., Sanchez P., Losada A. (2009). Epigenetic specification of centromeres by CENP-A. Exp. Cell Res..

[108] Black B.E., Bassett E.A. (2008). The histone variant CENP-A and centromere specification. Curr. Opin. Cell Biol..

[109] Black B.E., Jansen L.E., Foltz D.R., Cleveland D.W. (2010). Centromere identity, function, and epigenetic propagation across cell divisions. Cold Spring Harb. Symp. Quant. Biol..

[110] Choo K.H. (2001). Domain organization at the centromere and neocentromere. Dev. Cell.

[111] Henikoff S., Dalal Y. (2005). Centromeric chromatin: What makes it unique?. Curr. Opin. Genet. Dev..

[112] McKinley K.L., Cheeseman I.M. (2016). The molecular basis for centromere identity and function. Nat. Rev. Mol. Cell Biol..

[113] Sullivan B.A., Karpen G.H. (2004). Centromeric chromatin exhibits a histone modification pattern that is distinct from both euchromatin and heterochromatin. Nat. Struct. Mol. Biol..

[114] Nasmyth K., Haering C.H. (2009). Cohesin: Its roles and mechanisms. Annu. Rev. Genet..

[115] Kunitoku N., Sasayama T., Marumoto T., Zhang D., Honda S., Kobayashi O., Hatakeyama K., Ushio Y., Saya H., Hirota T. (2003). CENP-A phosphorylation by aurora-A in prophase is required for enrichment of aurora-B at inner centromeres and for kinetochore function. Dev. Cell.

[116] Wike C.L., Graves H.K., Hawkins R., Gibson M.D., Ferdinand M.B., Zhang T., Chen Z., Hudson D.F., Ottesen J.J., Poirier M.G. (2016). Aurora-A mediated histone H3 phosphorylation of threonine 118 controls condensin I and cohesin occupancy in mitosis. Elife.

[117] Godek K.M., Kabeche L., Compton D.A. (2015). Regulation of kinetochore-microtubule attachments through homeostatic control during mitosis. Nat. Rev. Mol. Cell Biol..

[118] Barisic M., Aguiar P., Geley S., Maiato H. (2014). Kinetochore motors drive congression of peripheral polar chromosomes by overcoming random arm-ejection forces. Nat. Cell Biol..

[119] Chmatal L., Yang K., Schultz R.M., Lampson M.A. (2015). Spatial regulation of kinetochore microtubule attachments by destabilization at spindle poles in meiosis i. Curr. Biol..

[120] Ye A.A., Deretic J., Hoel C.M., Hinman A.W., Cimini D., Welburn J.P., Maresca T.J. (2015). Aurora A kinase contributes to a pole-based error correction pathway. Curr. Biol..

[121] Kim Y., Holland A.J., Lan W., Cleveland D.W. (2010). Aurora kinases and protein phosphatase 1 mediate chromosome congression through regulation of CENP-E. Cell.

[122] Barisic M., Silva e Sousa R., Tripathy S.K., Magiera M.M., Zaytsev A.V., Pereira A.L., Janke C., Grishchuk E.L., Maiato H. (2015). Microtubule detyrosination guides chromosomes during mitosis. Science.

[123] Lampson M.A., Cheeseman I.M. (2011). Sensing centromere tension: Aurora B and the regulation of kinetochore function. Trends Cell Biol..

[124] Lampson M.A., Renduchitala K., Khodjakov A., Kapoor T.M. (2004). Correcting improper chromosome-spindle attachments during cell division. Nat. Cell Biol..

[125] Katayama H., Sasai K., Kloc M., Brinkley B.R., Sen S. (2008). Aurora kinase-A regulates kinetochore/chromatin associated microtubule assembly in human cells. Cell Cycle.

[126] DeLuca K.F., Meppelink A., Broad A.J., Mick J.E., Peersen O.B., Pektas S., Lens S.M.A., DeLuca J.G. (2018). Aurora A kinase phosphorylates Hec1 to regulate metaphase kinetochore-microtubule dynamics. J. Cell Biol..

[127] Deng Z., Cao P., Wan M.M., Sui G. (2010). Yin yang 1: A multifaceted protein beyond a transcription factor. Transcription.

[128] Gordon S., Akopyan G., Garban H., Bonavida B. (2006). Transcription factor YY1: Structure, function, and therapeutic implications in cancer biology. Oncogene.

[129] Alexander K.E., Rizkallah R. (2017). Aurora A phosphorylation of YY1 during mitosis inactivates its DNA binding activity. Sci. Rep..

[130] Chuang L.S., Khor J.M., Lai S.K., Garg S., Krishnan V., Koh C.G., Lee S.H., Ito Y. (2016). Aurora kinase-induced phosphorylation excludes transcription factor RUNX from the chromatin to facilitate proper mitotic progression. Proc. Natl. Acad. Sci. USA.

[131] Kabeche L., Nguyen H.D., Buisson R., Zou L. (2018). A mitosis-specific and R loop-driven ATR pathway promotes faithful chromosome segregation. Science.

[132] Castellano-Pozo M., Santos-Pereira J.M., Rondon A.G., Barroso S., Andujar E., Perez-Alegre M., Garcia-Muse T., Aguilera A. (2013). R loops are linked to histone H3 S10 phosphorylation and chromatin condensation. Mol. Cell.

[133] Holt S.V., Vergnolle M.A., Hussein D., Wozniak M.J., Allan V.J., Taylor S.S. (2005). Silencing CENP-F weakens centromeric cohesion, prevents chromosome alignment and activates the spindle checkpoint. J. Cell Sci..

[134] Marston A.L. (2015). Shugoshins: Tension-sensitive pericentromeric adaptors safeguarding chromosome segregation. Mol. Cell Biol..

[135] Daum J.R., Potapova T.A., Sivakumar S., Daniel J.J., Flynn J.N., Rankin S., Gorbsky G.J. (2011). Cohesion fatigue induces chromatid separation in cells delayed at metaphase. Curr. Biol..

[136] Stevens D., Gassmann R., Oegema K., Desai A. (2011). Uncoordinated loss of chromatid cohesion is a common outcome of extended metaphase arrest. PLoS ONE.

[137] Caous R., Pascal A., Rome P., Richard-Parpaillon L., Karess R., Giet R. (2015). Spindle assembly checkpoint inactivation fails to suppress neuroblast tumour formation in aura mutant *Drosophila*. Nat. Commun..

[138] Yu F., Jiang Y., Lu L., Cao M., Qiao Y., Liu X., Liu D., Van Dyke T., Wang F., Yao X. (2017). Aurora-A promotes the establishment of spindle assembly checkpoint by priming the Haspin-aurora-B feedback loop in late G2 phase. Cell Discov..

[139] Zhou L., Liang C., Chen Q., Zhang Z., Zhang B., Yan H., Qi F., Zhang M., Yi Q., Guan Y. (2017). The N-terminal non-kinase-domain-mediated binding of Haspin to Pds5b protects centromeric cohesion in mitosis. Curr. Biol..

[140] Thein K.H., Kleylein-Sohn J., Nigg E.A., Gruneberg U. (2007). Astrin is required for the maintenance of sister chromatid cohesion and centrosome integrity. J. Cell Biol..

[141] Chiu S.C., Chen J.M., Wei T.Y., Cheng T.S., Wang Y.H., Ku C.F., Lian C.H., Liu C.C., Kuo Y.C., Yu C.T. (2014). The mitosis-regulating and protein-protein interaction activities of Astrin are controlled by aurora-A-induced phosphorylation. Am. J. Physiol. Cell Physiol..

[142] Mersfelder E.L., Parthun M.R. (2006). The tale beyond the tail: Histone core domain modifications and the regulation of chromatin structure. Nucleic Acids Res..

[143] Nerusheva O.O., Galander S., Fernius J., Kelly D., Marston A.L. (2014). Tension-dependent removal of pericentromeric shugoshin is an indicator of sister chromosome biorientation. Genes Dev..

[144] Indjeian V.B., Stern B.M., Murray A.W. (2005). The centromeric protein Sgo1 is required to sense lack of tension on mitotic chromosomes. Science.

[145] Liu H., Jia L., Yu H. (2013). Phospho-H2a and cohesin specify distinct tension-regulated Sgo1 pools at kinetochores and inner centromeres. Curr. Biol..

